# Nursing Students’ Perception about Gender Inequalities Presented on Social Networks: A Qualitative Study

**DOI:** 10.3390/ijerph20031962

**Published:** 2023-01-20

**Authors:** Elena Andina-Díaz, María Isabel Ventura-Miranda, Enedina Quiroga-Sánchez, Ángela María Ortega-Galán, Isabel María Fernández-Medina, María Dolores Ruiz-Fernández

**Affiliations:** 1 Department of Nursing and Physiotherapy, University of León, Vegazana Campus, 24170 Leon, Spain; 2 SALBIS Research Group, University of León, 24402 Leon, Spain; 3 EYCC Research Group, University of Alicante, 03690 Alicante, Spain; 4 Department of Nursing, Physiotherapy, and Medicine, University of Almeria, Carretera San Urbano, 04120 Almería, Spain; 5 Department of Nursing, University of Huelva, Campus el Carmen, 21071 Huelva, Spain; 6 Facultad de Ciencias de la Salud, Universidad Autónoma de Chile, Providencia 4780000, Chile

**Keywords:** photovoice, gender stereotyping, COVID-19, nursing students

## Abstract

During the COVID-19 pandemic, gender inequalities in nurses have been exacerbated through the images shown on social networks. This study aimed to explore and describe nursing students’ experiences and perceptions about gender inequalities in nurses during the COVID-19 pandemic. A descriptive qualitative study was carried out in two universities in 264 undergraduate nursing students. The photovoice method was used to guide the study. Results: Two main categories and four subcategories were described from the data: “gender-related stereotypes”, with “male leadership in a female profession” and “sexualization of female nurses” and “women’s vulnerability in the pandemic” with “the gender gap in the face of increased risk of contagion “ and “women’s emotional fragility”. Over the years, care has been considered a female task, and nursing continues to be thought of in this way. The nurse has been discriminated against, poorly considered as a professional, and, as a woman, subjected to gender roles.

## 1. Introduction

The COVID-19 pandemic has become the most critical health crisis in recent times [1]. Nurses have been the main protagonists during its development [2]. They have been subjected to direct and continuous exposure to the virus in exceptional situations with a lack of human and material resources, as they have been on the front line of care [3]. In addition, they have faced major ethical and moral decisions and have had to balance patient care and their own family and personal needs [4]. The suffering and coping with the death of patients have placed nurses in an extreme situation, which has resulted in a series of repercussions on their emotional well-being [5].

Posting images on social networks has become a common way of showing the fear, stress, and fatigue that nurses have suffered [6]. In this regard, signs of suffering have been shown unequally between men and women during the pandemic [7]. Overall, 70% of COVID-19 infections of nurses in countries such as the U.S., Spain, and Italy are women [8]. In addition, the considerable feminization of nurses and the social role that women play in care have been accentuated [9]. Thus, stereotypes have mediated the expression of emotions between men and women in social networks, emphasizing gender inequalities. A gender stereotype is defined as “a generalized opinion or prejudice about attributes or characteristics that men and women possess or should possess, or the social roles that both perform or should perform” [10]. These stereotypes can be negative and harmful since they perpetuate an unreal image that does not correspond to the reality of the nursing profession [11]. In this way, the gender bias experienced by front-line nurses still greatly challenges their emotional and personal identity [12].

It is necessary for future nurses to approach the reality of the profession and to be aware of the existence of gender inequalities that have increased during the COVID-19 pandemic [13]. The photovoice method allows us to observe the daily reality and criticize it. The critical and analytical capacity of nursing students on certain aspects of the gender gap and stereotypes shown in photographs can increase the visibility of the nursing profession [14]. The stereotypical image of a nurse as a women’s job is a barrier to the professional development of nurses [15]. However, no research has been found that examines the knowledge that nursing students have about this latent reality.

Therefore, the aim of this study was to explore and describe nursing students’ experiences and perceptions about gender inequalities in nurses during the COVID-19 pandemic.

## 2. Materials and Methods

### 2.1. Design

A descriptive qualitative study was carried out. The photovoice method was used to guide the study [16]. This photographic technique allows people to observe their everyday realities [17,18] or the reality of others [19,20]. It can be used in academic context to stimulate critical thinking in students, to promote the search for and description of photographs, and to discuss the meanings behind these images. On this point, an educational activity was proposed, using and adapting the three photovoice phases [16]:

(i) Involving students in documenting society’s concerns with the photographs within social networks (Twitter^®^);
(ii) Engaging participants in a reflective process, whereby they critically discuss and analyze society’s discourses (tweets);
(iii) Disseminating participant-generated results within the community in a photography exhibition.

Twitter is one of the most popular microblogging services. It allows users to write short messages and broadcast them to their followers in real time. Compared to other more current networks, such as Instagram, this social platform allows the user to generate a digital discussion forum, to carry out conversations, and to inform and share news [21].

Nurses comprise one of the great users groups and assets of Twitter [22]. Through it, they expand the content beyond that broadcasted during a certain time and place, so it is relevant for perceiving many current events, especially related to health [23].

### 2.2. Sample and Settings

Participants were undergraduate nursing students enrolled in the 2020–2021 academic year in the required modules of Transculturality, Health, and Gender (1st year, University of León, Spain) and Community Nursing II (2nd year, University of Almería, Spain). They were selected for inclusion using convenience sampling [24]. The inclusion criteria were to be a nursing student and enrolled in these modules. Participants were excluded if they refused to participate in the study (none of them).

### 2.3. Procedures

Data were collected independently at the two universities, in two sessions. Firstly, a theoretical introduction to gender stereotypes, the aims of the activity (gender stereotypes in nurses shown in Twitter in first months (March and April 2020) of COVID-19 pandemic, the work to be carried out, and Twitter management were explained to the students. Each student had to collect three tweets (not related to institutions) and describe them using the SHOWED model (specific for photovoice) in which they asked to reflect on (i) what you See; (ii) what is really Happening; (iii) how does this relate to Our lives; (iv) Why things are this way and Why this happens; (v) how this image could Educate/empower people; and (vi) what we can Do about it [25]. Secondly (classroom/video conferencing), the students, assisted by their narratives/tweets (SHOWED model), discussed the gender stereotypes in nurses seen in Twitter during the first months of pandemic (photo-elicitation). The facilitators (two from each university) provided guidance and support to complement the inductive analytical process and recorded the students’ thoughts. Finally, the most relevant tweets were selected and exhibited for a month at the faculty/virtual space (photography exhibition) to induce critical debate in the university community.

### 2.4. Data Analysis

The data from the narratives/tweets (SHOWED models) were analyzed using thematic analysis to reflect reality and to unpick the surface [26]. This analysis consists of different phases: (i) becoming familiar with the data; (ii) generating initial codes; (iii) searching for themes; (iv) reviewing themes; (v) defining and naming themes; and (vi) producing the report. The COREQ checklist was used to check the quality of the results and guide the writing process [27]. A qualitative data-management package (MAXQDA2020^®^) was used to order the process of analysis.

The criteria of credibility, confirmability, dependability, and transferability were used to assure the rigor of the study. To confirm the interpretation made with the narratives, all the participants received a copy of the data. To demonstrate confirmability, a researcher who had no relationship with the participants conducted the interviews. The interpretation of the results was carried out by two researchers with the aim to assure dependability. In addition, a detailed description of the steps of the study and data collection was provided. Finally, transferability was approved by checking participants’ narratives [28].

### 2.5. Ethical Considerations

The students were informed regarding the voluntarily of the participation in the educational activity and the possibility to refuse or drop out without negative consequences. Moreover, they received a participant sheet to sign. The Ethics Committee of the two universities approved the study (ETICA-ULE-017-2021, EFM 180/2022).

## 3. Results

**Figure 1:**
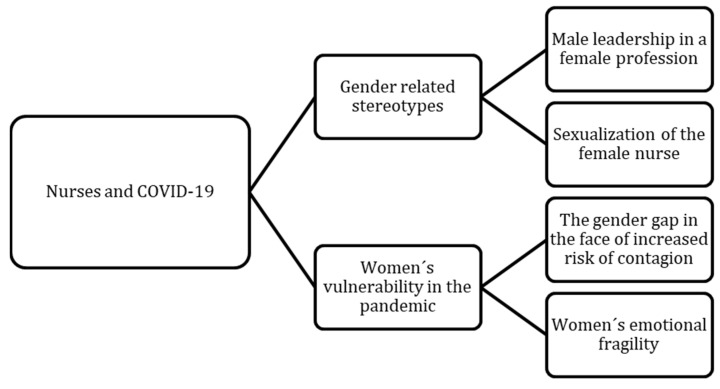
Matrix “Healthcare professionals and COVID”.

A total of two hundred and sixty-four nursing students (47 of them were men, and 217 were women) with an age between 18 and 24 years (88.3%) participated in the study (Table 1). The results were obtained from a qualitative evaluation of the photographs and narratives. From the data we can highlight two main categories and four subcategories (Figure 1). In addition, we show some representative photographs and significant units as examples (Table 2).

Considering the narratives of the students, gender inequalities were explicitly presented in social media during the COVID-19 pandemic.

### 3.1. Gender-Related Stereotypes

Since ancient times, care has been linked to the female sex. In consequence, the nursing profession is totally associated with the female gender and the field of care. This category explains how nursing students perceive the association between gender and professional category [29,30].

#### 3.1.1. Male Leadership in a Female Profession

According to the point of view of our participants and as shown on social networks, in pandemic times, most of the nurses’ leadership involved men. This occurs because when the profession enjoys a higher status, this stereotype is even stronger and is even normalized:

“Men nurses only constitute only 14% of the total nursing profession, while they occupy almost all managerial positions.”

(L, P55)

The students reflected on the fact of how to break these stereotypes since it is women who have fought the most against the pandemic and have borne the greatest emotional burden. Nurses during the pandemic have demonstrated sufficient leadership capacity to lead in representative positions:

“We should include women in everything related to health, since women have fought as much or even more against this virus since the number of women is very high.”

(L, P56)

“From my point of view, they have wanted to reflect how in this pandemic, women have gained more strength in the world of work and especially in the health sector, directing healthcare centers and assuming certain roles, because nursing is one of the professions with a higher percentage of women. While the husbands have stayed at home with the children taking care of them and playing the role of mother.”

(A, P5)

#### 3.1.2. Sexualization of the Female Nurse

In social networks, the sexualization of nurses has been shown through photographs. Photos of nurses with sexy and provocative uniforms, closely related to the gender stereotype, can be seen. This shows us that this perception continues to be rooted today, and despite the pandemic, the nurse is still seen as a sexy object in the visualization of her work [31,32]:

“This situation occurs due to the gender stereotypes that currently exist and the mentality of part of the population about women in the nursing profession.”

(A, P13)

“We see the people who take care of us as a sexy representation. Very stereotyped, when they are simply professionals doing their job.”

(L, P22)

Gender inequality is reflected in the images: while women are represented with images of weakness, men are represented as warriors or superior beings who fight against illness [33,34].

“All eyes have been fixed on the nurses, represented in countless posters, graffiti and illustrations. Most of them have one feature in common. While women are depicted with sad, tearful, or afflicted faces, men are seen as soldiers or fighters against disease. The gender roles that are so deep in our society.”

(L, P40)

### 3.2. Women’s Vulnerability in the Pandemic

The coronavirus has caused not only a very serious health emergency but also the aggravation of many pre-existing inequalities due to both the social and economic impact of the pandemic and to the measures adopted to alleviate it. In this scenario, women have suffered and continue to suffer particularly acutely the consequences of these inequalities, having to face greater vulnerability and new obstacles to achieving equality. The images collected by the students showed how the nurses were more vulnerable to the virus both physically (more risk of contagion) and emotionally (showing more weakness, expressing their feelings more).

#### 3.2.1. The Gender Gap in the Face of Increased Risk of Contagion

Through social networks, the participants perceived that during the pandemic, the number of COVID-19 infections among female nurses increased, as they were the ones who provided most of the care:

“Women are the highest percentage that provide care to sick people, as a result of the feminization of professions, this being a clear example, and thus increasing the negative consequences of this work on women, since they are more exposed to the disease than men.”

(L, P2)

“Women form the highest percentage that provide care to sick people, thus increasing the negative consequences of this work on women since they are more exposed to the disease than men.”

(A, P11)

This fact has been reported by the participants as another example of the gender inequality that exists in the nurses, which has been demonstrated worldwide.

“This is news that explains that the UN warns of the existence of the gender gap in the pandemic, making visible that 70% of the infected healthcare professionals are women.”

(L, P4)

#### 3.2.2. Women’s Emotional Fragility

During the confinement related to COVID-19, countless images uploaded on social networks by nurses could be seen, and most of these photographs, according to the participants, showed the woman, sad, crying, afflicted, and despondent, while the photos uploaded by the men showed men who were tired but never crying or sad:

“This image represents women as emotional, weak, with a lack of control over their emotions, who are not strong… since it is something that is socially imposed and accepted: women cry, men don’t.”

(A, P5)

The degree of suffering and helplessness is reflected in the photographs. Participants expressed the anguish experienced by nurses during the pandemic. The faces in the photographs showed the extreme and intense conditions that they had to endure at work, and even so, they continued to provide care despite the repercussions that this could have at the psychological and physical levels:

“After weeks of confinement and hard work on shifts in which she could not remove her PPE, and her colleagues had to give her food and drink because she could not afford to leave the emergency room, the nurse overwhelmed by all the stress, tension, uncertainty, indignation, pressure, sorrow, heat, hunger, dehydration, and exhaustion that she is suffering from.”

(A, P23)

The participants reported that most of the images are of women expressing their feelings, which is a fact assumed and normalized by society. For this reason, physical and emotional exhaustion is expressed through women. Women are even given the quality of being more compassionate, alleviating the suffering of others:

“We can see in the images several women who show great emotional and physical discomfort through the marks on their skin and their looks. We should especially normalize the fact that men also suffer (and more so in situations of such emotional burden as the pandemic) and avoid trying to only give the appearance of compassion to women.”

(L, P50)

Participants expressed that women lack the skills and attitudes to cope with suffering. In addition, they reported that they are not able to control and manage emotions in adverse situations. The participants attributed these shortcomings to the female gender, demonstrating a certain inequality:

“The nurse shows the physical and emotional exhaustion that she has suffered during those days of work. Many times, as women, we do not feel strong or sufficiently prepared to face challenges.”

(A, P8)

## 4. Discussion

The study explored nursing students’ experiences and perceptions about gender inequalities in nurses during the COVID-19 pandemic. The emergency caused by COVID-19 is posing a major challenge to nursing professionals. There is no doubt about the importance of this profession when it comes to fighting the virus, and nurses are seen as protagonists in the news and social networks [35].

In terms of stereotypes, the students reflected on very established images about gender roles. Even though the nursing profession is predominantly female, the idea of male versus female leadership was evident on social networks during the pandemic through representative tweets. This idea is present today, demonstrating nuances of a patriarchal system and showing us a scenario with inverted power relationships [36,37].

The students also highlighted the idea of the sexualization experienced by women, with traditional roles and stereotypes being placed on nursing. Images shown on Twitter, collected by students, show nurses wearing sexy uniforms. These types of images alter society’s perception of nurses. Nursing currently has a very limited role in the eyes of society. The nurse is always visualized as a woman and servile to the doctor, and it is clear that she is everything except a professional. As research shows us, limited and sexist stereotypes still exist [38,39]. A study carried out by Domínguez and Sapiña (2021) collected the visual representation that nurses have had about COVID-19, highlighting how nursing is represented with a stereotyped role in which nurses (female) act as a companion to the doctor (male), as “guardian angels” dressed in miniskirts or with low necklines, etc. Our profession is still highly conditioned by sexist stereotypes that distort our image in society and undermine the importance of the profession [40].

The students focused on the “helplessness” experienced by nurses as the second category. The nurse is in direct contact with patients with suspected and/or confirmed coronavirus infection, putting their lives and those of their loved ones at risk. Despite efforts, gender roles define care as a task almost exclusively performed by women. In this way, women support the fundamental burden of the care system both formally and informally [41].

The crisis triggered by the COVID-19 pandemic has a significant gender impact in this sphere, placing care at the center and women as the fore of the initial response to the disease. Nursing, in addition to the usual difficulties of conciliation and work overload, must combine the co-responsibility of protecting others and protecting oneself [42,43].

The students reflected on the helplessness that nurses have suffered in times of pandemic. In relation to the increase in exposure, women represent 70% of healthcare professionals, reaching 84% in the case of nurses [44]. During this pandemic period, nursing, with direct and continuous contact with people affected by coronavirus, has made visible the importance, pressure, and burden of care and has shown the need for both care and the focus on gender to be part of the social strategies of countries [45,46]. The International Council of Nurses (ICN) has denounced the unacceptable inequalities of the profession that the COVID-19 pandemic has brought to light. Nurses present a double inequality: social status and gender [47].

The students highlighted the increased emotional impact on the figure of the nurse. Stress, work overload, and the confrontation of feelings take a toll on the coping skills of nurses [48]. The female nurses compared to male nurses appear emotionally weak. Since the outbreak of the virus within the health system, various studies have shown how emotions are highly mediated by stereotypes traditionally associated with men and women [49]. This is in line with previous studies. Aranda et al. defined how stereotypes about men and women not only affect the perception that other people have about nursing but also the roles that they must play based on these stereotypes.

These results support the existing gender inequalities in the profession and their growth during COVID-19 pandemic [50,51,52]. The sexualization of nurses undermines the social vision of the profession. Care has been associated with the female sex, and stereotypes also affect society’s perception of nursing. During the pandemic, nurses have endured a heavy workload that has affected their physical and emotional health.

### Limitations

The different discourses that emerged in this study may have been limited mainly by the homogeneity of the sample. Firstly, the study carried out with nursing students, with a greater percentage of them being female. However, this is normal since most nursing students are women. Secondly, the participants are first- and second-year students who have not completed internships. Their perception may be totally different from students who are in higher courses. Thirdly, the age of the participants was similar, which could have influenced their perception. Nursing students’ perceptions in other geographical locations or countries could differ. Future research should be developed with nursing students from different geographical locations. However, this research is the only study to date that explores gender inequalities in nurses during the pandemic in Spain. Finally, this study only reflects the experiences of nursing students. Future research should include the point of view of health science students and healthcare professionals.

## 5. Conclusions

Nursing students explored gender inequalities in nurses during the pandemic through their thoughts about Twitter images, and reflecting on the burden that gender has carried and still carries in the profession. Care has always been considered a female task, and nursing has continued to carry this conception. The nurse is in a situation of discrimination as a professional, where she is given little consideration and, as a woman, where she is subject to gender roles. The health crisis has further emphasized these existing gender inequalities. Nurses continue to face numerous gendered challenges daily. The elimination of gender stereotypes in nursing is necessary for the profession to have the recognition, opportunities, and place it deserves in society.

However, this study contains helpful information. It is indispensable to work with students on the visibility of the nursing profession in clinical practice through social networks. This would allow them to work with students on gender equality and give visibility to the work and competences of nurses in society.

## Figures and Tables

**Table 1:** Characteristics of the participants (N = 264).

	N	%
Age (years)		
18–24	233	88.3
25–34	19	7.2
35–44	6	2.3
45–54	4	1.5
>55	2	0.8
Sex		
Male	47	16
Female	217	84
University		
Almería	115	43.6
León	149	56.4
Course		
First year	149	56.4
Second year	115	43.6

**Table 2:** Categories, subcategories, and representative photographs/significant units highlighted from the data.

Categories	Subcategories	Some Representative/Significant Units
Gender-related stereotypes	Male leadership in a female profession	* 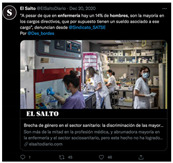 * (L, P55) Normalized micro sexism
		*Translation of tweet: “Despite the fact that in nursing there are 14% of men, the majority are nurses in management positions, who of course have a salary associated with that position” denounces the @SÁSTE trade union. By @Des_bordes
		“Gender gap in the health sector: discrimination against female nurses in the…” “They are more than half of the medical profession, and an overwhelming majority in nursing and the socio-health sector, but this fact has not managed to…”
	Sexualization of the female nurse	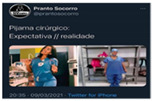 * Translation of tweet: Second tweet: Surgical pyjamas Expectation/reality (A, P13) Sexy representation of the profession
Women’s vulnerability in the pandemic	The gender gap in the face of increased risk of contagion	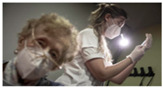 (L, P11) Gender gap
	Women’s emotional fragility	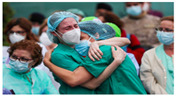 (A, P5) More emotional women
